# Sparse Independence Component Analysis for Competitive Endogenous RNA Co-Module Identification in Liver Hepatocellular Carcinoma

**DOI:** 10.1109/JTEHM.2023.3283519

**Published:** 2023-06-07

**Authors:** Yuhu Shi, Lili Zhou, Weiming Zeng, Boyang Wei, Jin Deng

**Affiliations:** Information Engineering CollegeShanghai Maritime University 12477 Shanghai 201306 China; Yangpu District Central Hospital Shanghai 200433 China; College of Mathematics and InformaticsSouth China Agricultural University 12526 Guangzhou 510642 China

**Keywords:** Sparse ICA, lncRNA, ceRNA, co-expression modules, LIHC

## Abstract

Objective: Long non-coding RNAs (lncRNAs) have been shown to be associated with the pathogenesis of different kinds of diseases and play important roles in various biological processes. Although numerous lncRNAs have been found, the functions of most lncRNAs and physiological/pathological significance are still in its infancy. Meanwhile, their expression patterns and regulation mechanisms are also far from being fully understood. Methods: In order to reveal functional lncRNAs and identify the key lncRNAs, we develop a new sparse independence component analysis (ICA) method to identify lncRNA-mRNA-miRNA expression co-modules based on the competitive endogenous RNA (ceRNA) theory using the sample-matched lncRNA, mRNA and miRNA expression profiles. The expression data of the three RNA combined together is approximated sparsely to obtain the corresponding sparsity coefficient, and then it is decomposed by using ICA constraint optimization to obtain the common basis and modules. Subsequently, affine propagation clustering is used to perform cluster analysis on the common basis under multiple running conditions to obtain the co-modules for the selection of different RNA elements. Results: We applied sparse ICA to Liver Hepatocellular Carcinoma (LIHC) dataset and the experiment results demonstrate that the proposed sparse ICA method can effectively discover biologically functional expression common modules. Conclusion: It may provide insights into the function of lncRNAs and molecular mechanism of LIHC. Clinical and Translational Impact Statement–The results on LIHC dataset demonstrate that the proposed sparse ICA method can effectively discover biologically functional expression common modules, which may provide insights into the function of IncRNAs and molecular mechanism of LIHC.

## I. Introduction

Long noncoding RNAs (lncRNAs) refer to RNA transcripts with a length of more than 200 nucleotides and no significant protein-coding ability [1], which were once regarded as a kind of transcription “noise” RNA with no biological functions. However, in recent years, more and more studies have shown that many lncRNAs are not transcriptional noises but major regulatory factors that affect the expression levels of dozens or even hundreds of target genes and play an important role in various biological processes such as transcription, splicing and translation, especially in a variety of tumors [2], [3], [4]. For example, lncRNA can be used as a biomarker for the diagnosis and prognosis of lung adenocarcinoma [5]. Compared with protein-coding genes, lncRNAs show superior potential in diagnosis and prognostic markers.

In addition, lncRNA is also believed to be involved in the pathogenesis of many diseases, including liver cancer [6], [7], [8]. For example, the lncRNA HULS has been found to be involved in tumorigenesis and serves as an endogenous sponge that inhibits the miRNA-372 and reduces miRNA-372-mediated translational repression of PRKACB in liver cancer [9]. BCYRN1 was found to regulate some cancer-related pathways through the lncRNA-miRNA-mRNA network and promote the occurrence of hepatocellular carcinoma (HCC), thus providing a new perspective for exploring the pathogenesis of HCC as a potential diagnostic and prognostic biomarker [10]. LncRNA KCNQ1OT1 has been found to play an important role in tongue squamous cell carcinoma (TSCC) growth and chemotherapeutic resistance and can be used as a new target for the treatment of TSCC [11]. However, due to the rapid development of lncRNAs, the role of lncRNA-related activities and their corresponding module patterns in physiological and pathological conditions remains unclear.

There are many known mechanisms by which lncRNA can play a role. Among them, more and more information show that lncRNA is involved in regulating tumor progression and tumor biological behavior through interaction with miRNAs or mRNAs [12], [13], [14]. In 2011, Salmena et al. proposed a competing endogenous RNA (ceRNA) hypothesis, which described a complex post-transcriptional regulatory network, including lncRNA, mRNAs and other types of RNA [15]. LncRNAs interact with miRNAs through miRNA binding sites (MREs) to regulate gene expression, and several experimental studies have confirmed this hypothesis [16], [17], [18]. For instance, Zhou et al. described the gene regulation by lncRNA-miRNA-mRNA ceRNA network in the progression TSCC, and proposed a new lncRNA-associated ceRNA that could help in the diagnosis and treatment of TSCC [19]. In addition, a large amount of evidence indicates that ceRNA has crosstalk in various cellular behaviors, and its disturbance can lead to the occurrence of diseases [20].

Although thousands of lncRNAs have been found and documented in public databases such as GENCODE, NONCODE and LNCipedia, the functional characterization of lncRNAs is still in its infancy. So far, only a few lncRNAs have been well functional commented [21], [22]. Considering the large number of lncRNAs and limited knowledge, we expected that functionally related lncRNAs would normally be associated with functionally related mRNAs or miRNAs, which have been demonstrated in several diseases [23], [24], [25], but most have not yet been functionally characterized. It is extremely expensive and laborious to experimentally determine the functional role of lncRNA in cancer. Therefore, it is very important to study the functional properties and tumor-specific lncRNA expression patterns of lncRNA by computational methods.

In this study, we proposed a new sparse ICA method to identify the co- modules of three RNAs, including mRNA, miRNA and lncRNA on the same set of samples. Firstly, the expression matrix of the three RNA combined together was approximated sparsely to obtain the corresponding sparsity coefficient, and then it was decomposed by using ICA constraint optimization to obtain the common basis and modules. Subsequently, affine propagation clustering (APC) was used to perform cluster analysis on the common basics under multiple running conditions to obtain the co- modules for the selection of different RNA elements. Finally, we evaluated the performance of this method and the results demonstrated its validity for the three RNA data analysis, which had a high correlation between the decomposition results and the original RNA expression data.

Liver Hepatocellular Carcinoma (LIHC) is one of the most common cancers in the world. Although great progress has been made in the research of liver cancer in recent years [26], [27], [28], its mechanism is still unclear, and novel and more effective biomarkers need to be explored for early diagnosis. Therefore, we applied the proposed sparse independence component analysis (ICA) to analyze three types of RNA data in LIHC patients, committed to identifying the differential expression of lncRNAs, miRNAs and mRNAs in LIHC, and construct a ceRNA network, so as to reveal their potential interaction in LIHC, and to find new targets and pathways for the development of therapeutic methods and the prolonging of patients’ survival time. The results suggested that specific lncRNAs were related to the occurrence and development of LIHC, in which 6 lncRNAs were significantly related to LIHC patient survival, which could be used as potential diagnostic biomarkers and therapeutic targets for LIHC.

## II. Materials and Methods

In this section, the relevant knowledge and detailed calculation process for sparse ICA and module elements selection will be described, respectively.

### A. Data Preparation

The LIHC transcript data and miRNA sequencing data were downloaded from The Cancer Genome Atlas (TCGA) database (https://cancergenome.nih.gov/) and then isolated lncRNA and mRNA data from the transcript data. Considering the method used in this study required that the three types of RNA data had the same dimensionality, that is, the number of samples corresponding to the three types of RNA data was the same. We systematically collected 20060 mRNAs, 1448 miRNAs and 7305 lncRNAs across 374 tumor samples and 50 control samples, which were denoted as three expression matrices. The detailed clinical information is showed in TABLE 1.

**TABLE 1:** Clinical Information of Patients

Clinical Parameters	LIHC	Control
Age, years (Mean ± SD)	61.7 ± 16.1	59.5 ± 13.5
Gender, n(%)		
Male	28 (56%)	253 (67.6%)
Female	22 (44%)	121 (32.4%)
Status, n(%)		
Alive	16 (32%)	243 (65.0%)
Dead	34 (68%)	130 (34.8%)
Unknown	0	1 (0.2%)

Note: SD, standard deviation; n, number.

### B. Independent Component Analysis

ICA is a technology developed in the study of blind signal separation, which assumes that the observed signals $X=\left ({{x_{1},x_{2},\cdots,x_{m}} }\right)^{\prime }$ is a linear mixture of independent source signals $S=\left ({{s_{1},s_{2},\cdots,s_{n}} }\right)^{\prime }$. Then the ICA model can be expressed as

\begin{equation*} X=A\cdot S \tag{1}\end{equation*}

where $A$ denotes a mixing matrix that mixes the independent source signals to generate the observed signals. The goal of ICA is to estimate an unmixing matrix $W$ such that $Y=\left ({{y_{1},y_{2},\cdots,y_{n}} }\right)^{\prime }$ is a good approximation to the true sources S.

\begin{equation*}Y=W\cdot X \tag{2}\end{equation*}

The most commonly used ICA algorithms include Informax, FastICA and so on [29].

### C. Affine Propagation Clustering

The specific framework of basic APC algorithm is presented in [30], which uses each data point as a potential cluster center, and then computing the similarity between each pair of data points. There are two kinds of message transmitted between data points: one is called “responsibility” $r\left ({{i,k} }\right)$, which represents the fitness of data point $x_{i}$ as centroid of data point $x_{k}$; and the other is availability $a\left ({{i,k} }\right)$, which refers to the degree of data point $x_{i} $ chooses data point $x_{k} $ as its centroid.

Generally, if the sum of the attractiveness of a data point to other data points and the sum of the belongingness of other data points to this point is relatively large, then the data point is more likely to become a centroid. On the contrary, if the sum of the attractiveness of data points to other data points and the sum of the belongingness of other data points to this point is relatively small, then the probability of this point becoming a centroid is also relatively small.

### D. Sparse Ica

In this study, the sparse ICA method is proposed to identify the co- modules of mRNA, miRNA and lncRNA of 374 tumor samples, in which the three RNA expression profiles are respectively denoted as $X_{1}$, $X_{2} $ and $X_{3}$ for the same set of samples. As a data-driven matrix decomposition method, ICA is a blind source separation method based on higher order statistical moments. The purpose of ICA is to decompose observed multivariate data into statistically independent and non-Gaussian source components. It has been widely used in mining and studying independent source components in various signal analysis. However, few studies have been directly applied to RNA gene expression data analysis. This study is aiming to identify competitive endogenous RNA common modules related to liver cancer from lncRNA-mRNA-miRNA data. From the perspective of data analysis, this is a matrix decomposition problem. For example, NMF method has been widely used in the analysis process of common modules. Therefore, ICA can be used to mine the common module information coefficient matrix of genes. In the following, they are cascaded as $X=\left ({{X_{1},X_{2},X_{3}} }\right)$ along the RNA expression dimension, and then it can be decomposed into the common basis matrix $A$ and module matrix $S=\left ({{S_{1},S_{2},S_{3}} }\right)$ according to the ICA definition as follows:

\begin{equation*} \left ({{X_{1},X_{2},X_{3}} }\right)=A\cdot \left ({{S_{1},S_{2},S_{3}} }\right) \tag{3}\end{equation*}

where $S_{1} $, $S_{2} $ and $S_{3}$ represent the corresponding module matrices for mRNA, miRNA and lncRNA, respectively. Each column in $A$ corresponds to each row in $S$. Because $A$ and $S$ in (1) are unknown, their analytic solutions cannot be obtained. Therefore, the goal is to obtain an inverse matrix $W$ of $A$ by using the constraint optimization method according to the independence assumption in the process of ICA solution, so that $\hat {S}=W\cdot X$ is a better approximate solution to $S$.

In addition, compared with all the genes that can be obtained, the dominant expression genes in the pathway corresponding to the common module are very few, thus presenting a certain sparsity, indicating that sparsity may be more consistent with the essential attribute of gene expression. As the most representative linear representation method of data, sparse representation has been successfully applied in the field of signal processing. At the same time, some researches show that it can significantly improve the analysis performance of ICA when considering sparsity in the calculating process [31]. Moreover, it is useful to obtain easily interpretable solutions by incorporating sparse constraints into decomposition of the RNA profile matrix [32]. Therefore, sparse approximation is firstly implemented in the proposed sparse ICA algorithm, which is used to obtain the sparse approximation coefficients. Based on the framework of sparse representation with a given dictionary $\Phi $, the sparse approximation process of observation data $X$ is expressed as:

\begin{equation*}X=C_{X} \cdot \Phi \tag{4}\end{equation*}

where the dictionary $\Phi $ can be a set of bases on the data space, such as wavelet bases or Fourier bases, and also can be gained through different algorithms on $X$ training, such as K-SVD. Then the sparse approximation process of module matrix $S$ under $\Phi $ can be expressed as

\begin{equation*}S=C_{S} \cdot \Phi \tag{5}\end{equation*}

According to the sparse theory, we can obtain the following formula from (3), (4) and (5) when an appropriate dictionary $\Phi $ is determined, namely:

\begin{equation*}C_{X} \approx A\cdot C_{S} \tag{6}\end{equation*}

The wavelet analysis method is adopted to obtain sparse expression in this study [33], including wavelet tree node decomposition, sparsity measuring, sparsity quality and optimal sparse nodes selection. Because only the sparse coefficient $C_{X} $ of $X$ can be obtained in the situation that only $X$ and dictionary $\Phi $ are known, so that (6) is the classical problem of blind source separation. Therefore, the solving of blind source separation problem of RNA expression profiles data $X$ in (3) is converted to the solving of blind source separation problem of the sparse approximation coefficients $C_{X}$ in (6), which can be modeled in the ICA framework as a constrained optimization problem as follows:

\begin{align*}&\textit {Maximize} J\left ({w }\right)\approx \left \{{{E\left [{ {G\left ({w }\right)} }\right]-E\left [{ {G\left ({v }\right)} }\right]} }\right \}^{2} \\&\textit {Subject to} h\left ({w }\right)=E\left [{ {w^{2}} }\right]-1=0 \tag{7}\end{align*}

where $w$ is a column vector of $W$, $J\left ({w }\right)$ is the contrast function used to measure the independence, and the approximation of negentropy is used as $J\left ({w }\right)$ in this study. $E\left [{ \cdot }\right]$ denotes the expectation operator. $G\left ({\cdot }\right)=log\left ({{cosh\left ({v }\right)} }\right)$ is a non-quadratic function, in which $v$ is a Gaussian random variable. The equality constraint $h\left ({w }\right)$ is used to compel the output signal have a unit covariance. To solve this optimization problem, the inequality constraint is transformed into equality constraint, $\hat {g}\left ({w }\right)=g\left ({w }\right)+c=0$ via introducing a slack variable $c$. Then, the augmented Lagrange method is utilized to search for the solution by fixed-point learning algorithm for the optimization problem (7), and the detailed solving process can be found in [34].

### E. Modules Elements Selection

Once obtaining the inverse matrix W, the common basis A and module matrix S are obtained. In order to obtain stable expression elements of the three types of RNA in this study, the sparse ICA method mentioned above is first running 20 times on the three types of RNA expression profiles data for the same samples, and then the corresponding basis matrixes and module matrixes are obtained for each time, respectively. Next, APC is used to perform cluster analysis on the basis matrixes of 20 times, in which the column vectors of each matrix used as the input data, and the centroid labels are obtained. According to the correspondence between columns of basis matrix and rows of module matrix in ICA, the row co- modules corresponding to the coefficient matrix S are obtained according to the centroid label in the cluster results.

**FIGURE 1.**
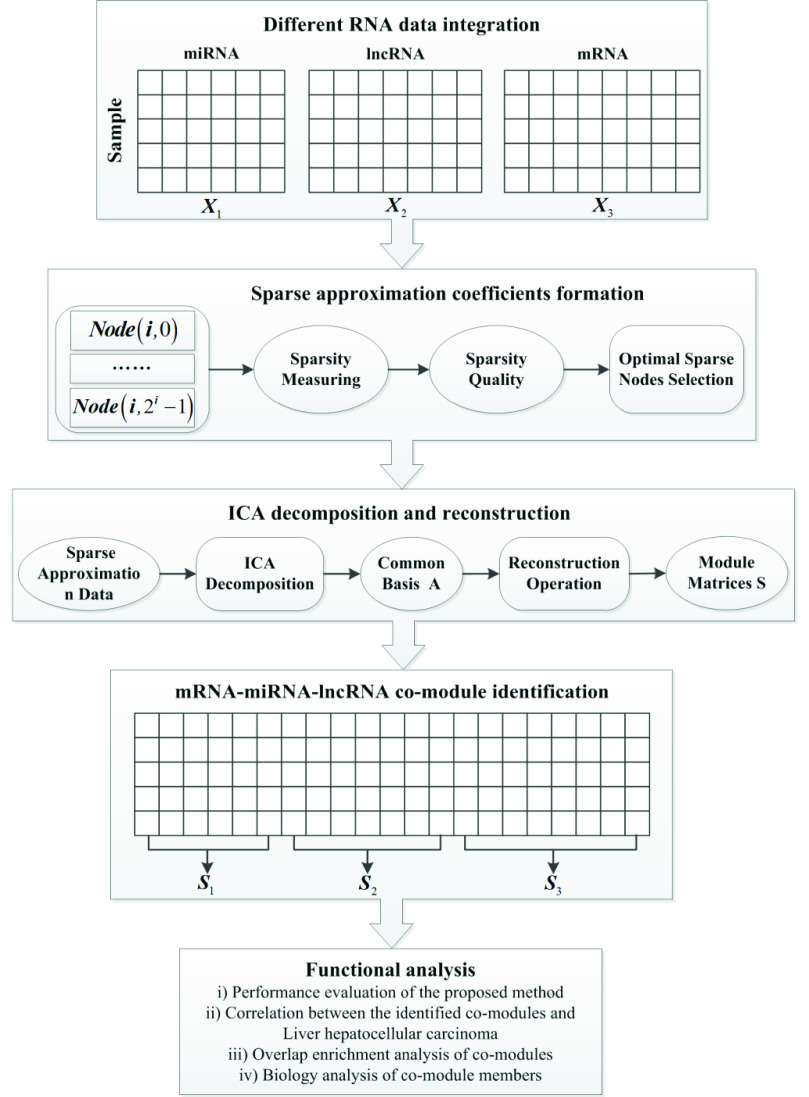
The flowchart of the sparse ICA method included different RNA data integration, sparse approximation coefficients formation and ICA decomposition and reconstruction, as well as co- module identification and functional analysis in LIHC.

Specifically, the number of modules used in the LIHC data analysis in this study is 40, so that the basis matrixes of 20 times contain $20\ast 40=800$ column vectors used as the input data in the cluster analysis. Then, two clusters are obtained through APC, and each cluster represents a co- module. When each co- module is z-scored, the threshold value of 0.05 is used to select the significance RNA elements corresponding to three types of RNA elements in each co- module, and then the intersection of significance RNA elements corresponding to different clusters is further calculated as the final result for the follow-up functional analysis. The analysis steps of this study introduced in Section II-B and II-C are shown in Fig. 1.

## III. Results and Analysis

In this section, we will first present the performance evaluation results of the proposed sparse ICA method, and then present the RNA analysis results of patients with LIHC.

### A. Performance Evaluation of Sparse Ica

**FIGURE 2.**
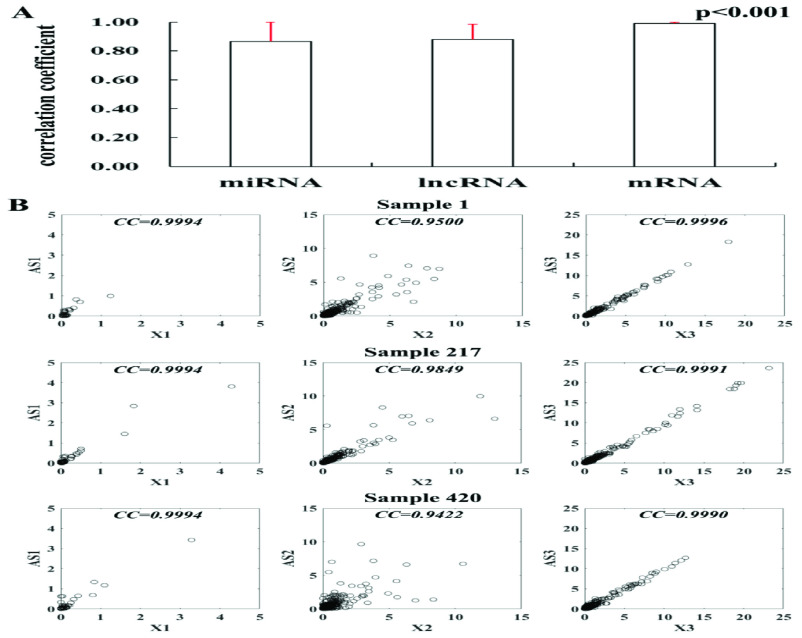
The evaluation results of sparse ICA performance. A) Histogram of sample-wise correlations of original and reconstructed miRNA, lncRNA and mRNA profiles across 424 samples, and the red line represents the standard deviation; B) Original data are plotted against the reconstructed miRNA, lncRNA and mRNA profiles with their correlation coefficients for three samples.

In order to better understand the relationship between different RNAs in LIHC, three types of RNA raw count data after normalization from LIHC samples were used as the input data of sparse ICA decomposition, and obtained 40 modules. Afterwards, the histogram of sample-wise correlations of original and reconstructed miRNA, mRNA and lncRNA profiles across 424 samples were constructed to evaluate the performance of the proposed sparse ICA method, as shown in Fig. 2.

For these 40 modules, we first calculated the correlation between the products of $AS_{i} \left ({{i=1,2,3} }\right)$ after decomposition and the original RNA data matrix $X_{i} \left ({{i=1,2,3} }\right)$. The average correlations of miRNA, lncRNA and mRNA were 0.87, 0.88 and 0.99 respectively, as shown in Fig. 2 A. On this basis, after randomly selecting three RNA data from three samples, we plotted the correlation between the reconstructed matrix and the original matrix in Fig. 2 B. It is obvious that the differences between the reconstruction matrix and the original matrix were small, which proved the robustness and effectiveness of the proposed sparse ICA method.

### B. Inferring Biological Functions for Co-Module

**FIGURE 3.**
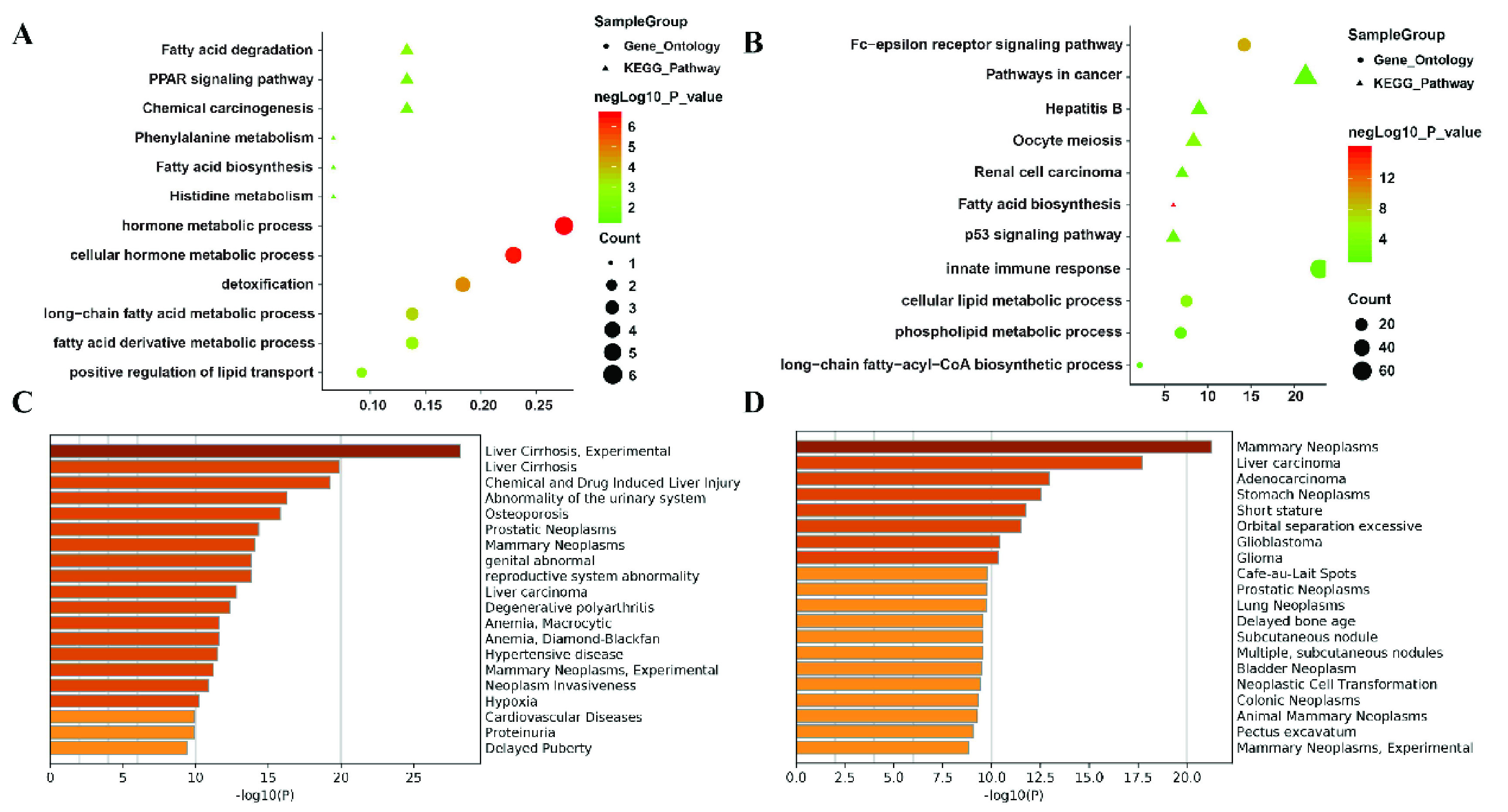
Biological function enrichment of co- module. A) KEGG pathway enrichment analysis and GO enrichment analysis of DE mRNAs. The horizontal axis represents the gene ratio in the enriched pathway or GO biological process. Circle nodes and triangle nodes represent GO and KEGG pathway, respectively. The size of nodes denotes the number of genes in enrichment sets and the color of nodes denotes the significance of results; B) KEGG pathway enrichment analysis and GO enrichment analysis of DE coding genes regulated by DE miRNAs. Row represents the average number of genes regulated by each RNA. C) Disease enrichment analysis of DE mRNAs. The horizontal axis and the color of bar denote the significance of results. D) Disease enrichment analysis of mRNAs regulated by DE miRNAs. The horizontal axis and the color of bar denote the significance of results.

The co- module included 395 mRNAs, 1350 lncRNAs and 251 miRNAs. In order to verify whether the co- module is related to cancer or LIHC, those differentially expressed (DE) mRNAs and miRNAs with P value less than 0.05 and $\vert $log2FC $\vert $ more than 2 are applied to perform the Kyoto Encyclopedia of Genes and Genomes (KEGG) enrichment analysis and Gene ontology (GO) enrichment analysis using the clusterProfiler R package [35] and DIANA-miRPath v3.0 software [36], where several significant pathways and biological processes with P value less than 0.05 shown in Fig. 3A and Fig. 3B. Moreover, DisGeNET [37] is applied to perform the disease enrichment analysis on DE mRNAs and mRNAs regulated by DE miRNAs, as shown in Fig. 3C and Fig. 3D.

The enrichment analysis revealed that the identified co- module is significantly enriched in a variety of KEGG pathways and GO terms, some of them have been reported to be involved in liver cancer. The single most striking observation to emerge from the figures is that both mRNA and miRNA are enriched in the pathways or biological processes related to fatty acid, such as fatty acid degradation, fatty acid biosynthesis, long-chain fatty acid metabolic process, etc. The previous study has found that cholesterol biosynthesis supports the growth of hepatocarcinoma lesions depleted of fatty acid synthase in humans [38]. The coding genes targeted by DE miRNAs are also enriched in pathways in cancer and p53 signaling pathway, all of which have been proved to be closely related to the growth of liver cancer cells [39]. Further analysis of disease enrichment reveals that the top rankings are tumor-related diseases and liver-related, such as liver cirrhosis, liver carcinoma and neoplasms, etc. These findings suggest the effectiveness of sparse ICA in identifying LIHC-specific co- modules that involved in multiple cancer-related cellular processes and signaling pathways.

### C. Cerna Network Construction Via Differentially Expressed Lncrnas

**FIGURE 4.**
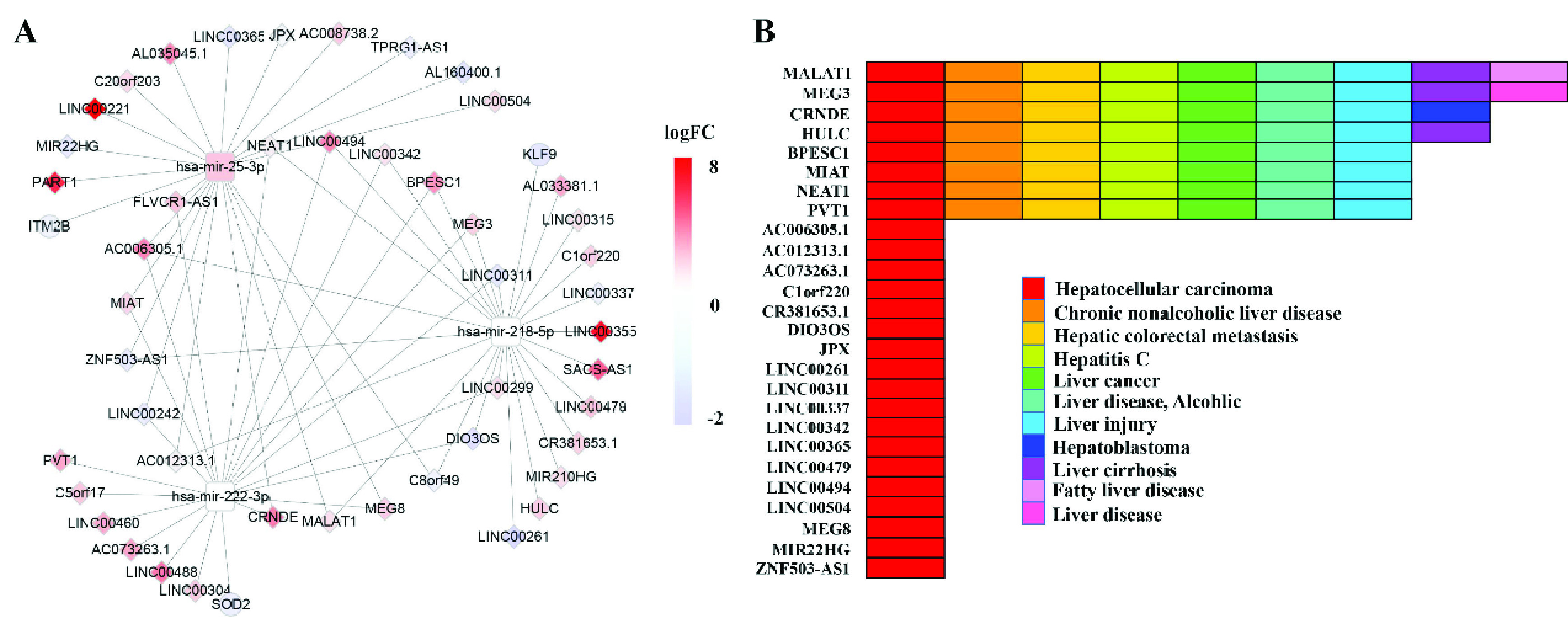
A) CeRNA network constructed by using the co- module, the color of nodes represents the expression change of RNA compared to control samples, and Red (blue) indicates a significant increase (decrease). Round rectangle node represents miRNA, ellipse node denotes mRNA and diamond node represents lncRNA. The edge denotes the regulatory relationship between the two types of RNA; B) The association of lncRNAs and liver-related diseases from lncRNA disease database.

So far, we know that many lncRNAs have been found during tumor development, but it is not clear what its function is. This study aims to use the ceRNA network to help researchers better understand the interaction mechanisms between lncRNAs and two other RNAs. The ceRNA network mainly explores the regulation and competition relationship of differential molecular composition. We first calculate significantly differentially expressed lncRNAs, mRNAs and miRNAs using the Deseq2 R package [40], and then use the mircode database [41] to find all the matching information for DE lncRNA, that is, miRNAs relate to DE lncRNAs. In order to find the targeted genes related to differentially expressed miRNAs, the starbase database [42] is applied to perform 3p and 5p annotation on miRNAs. For the labeled miRNAs, the corresponding regulatory genes are matched from three databases, including miRDB [43], miRTarBase [44] and TargetScan [45]. Finally, the ceRNA network related to DE lncRNAs is constructed using the relationship between three types of RNAs, as shown in Fig. 4A. Furthermore, in order to verify whether these lncRNAs in ceRNA are related to liver disease, the lncRNA-disease information is downloaded from the lncRNA disease v2.0 [46]. Interestingly, 26 lncRNAs can be found in this database, all of which are associated with liver-related diseases, as shown in Fig. 4B.

In this study, ceRNA includes 46 lncRNAs, 3 mRNAs, 3miRNAs, where all lncRNAs, mRNAs and 1 miRNA are significantly differentially expressed. Previous studies suggested that transfer of miR-25-3p by CHB-PNALT-Exo promoted the development of liver cancer by inhibiting the co- expression of TCF21 and HHIP [47]. Although there is no significant difference between the expression levels of miRNA-222 and miRNA-218, recent studies have demonstrated that miR-222 is a potential target in the quest for a cure for human liver fibrosis, and MNX1-AS1 promoted the malignant properties of HCC through targeting miR-218-5p/COMMD8 pathway [48], [49]. In addition, the previous study suggested that KLF9 significantly increased p53 stability in hepatocellular carcinoma cells and pharmacological or genetic activation of KLF9 may have potential in the treatment of LIHC [50]. The iTRAQ-based proteomics also reveals SOD2 as a potential salivary biomarker in liver cancer [51]. Moreover, most lncRNAs are associated with LIHC. For example, a previous study showed that HULC acted as a competing lncRNA to sequester miR-186 and thereby relieved miR-186-mediated HMGA2 repression in liver hepatocellular carcinoma [52]. The plasma MALAT1 level is associated with liver damage, and has clinical utility for predicting the development of liver hepatocellular carcinoma [53]. The shreds of evidences reveal the elements of ceRNA constructed by the co- module are closely associated with LIHC.

### D. Cox Regression and Survival Analyses for Cerna Identified by Co-Module

**FIGURE 5.**
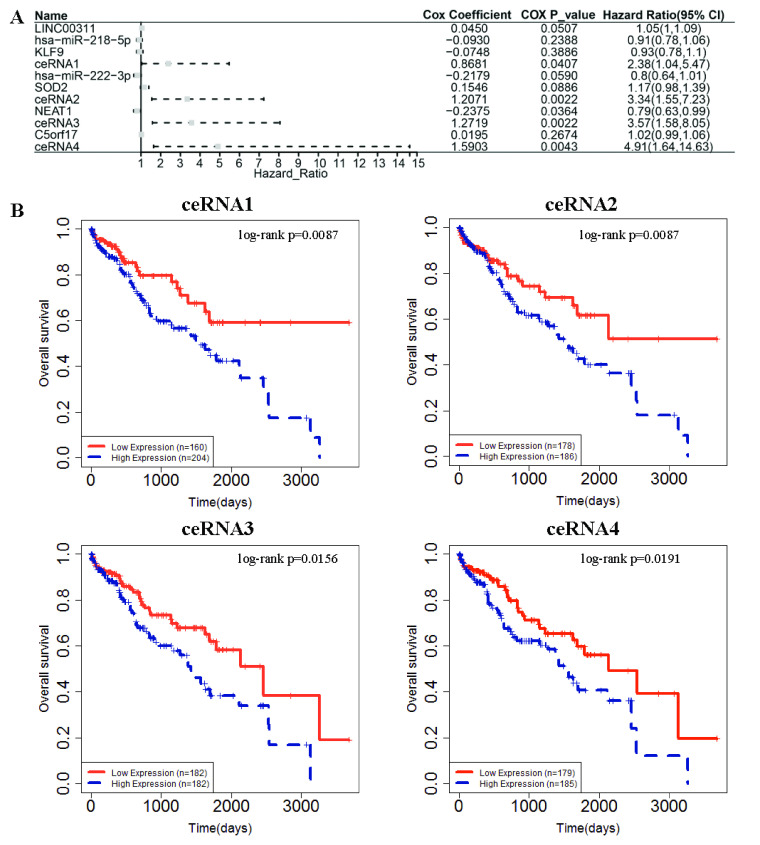
The survival status of ceRNA interaction in LIHC. A) Forest plot of multivariable Cox regression analysis. The boxes on the transverse lines show the hazard ratio (HR), and the transverse lines represent 95% confidence interval (CI); B) Kaplan-Meier survival analysis for ceRNA interaction. CeRNA1 denotes the interaction of LINC00311, hsa-miR-218-5p and KLF9; CeRNA2 denotes the interaction of LINC00311, hsa-miR-222-3p and SOD2; CeRNA3 denotes the interaction of NEAT1, hsa-miR-222-3p and SOD2; CeRNA4 denotes the interaction of C5orf17, hsa-miR-222-3p and SOD2.

The above analysis has revealed that the identified ceRNA co- module is closely associated with liver hepatocellular carcinoma. Hence, the co- module can be looked upon as a signature to inquire into the prognostic value of lncRNAs. The LnCeVar website [54] is applied to study the survival status of ceRNA interaction. After performing cox regression analysis and Kaplan-Meier survival analysis on all interactions of RNAs in ceRNA network, we find that 6 lncRNAs are significantly related to survival LIHC patient survival, including LINC00311, ZNF503-AS1, C5orf17, NEAT1, MEG3 and LINC00242. Also, all lncRNAs have proved to be associated with LIHC in previous analysis. Surprising, three lncRNA-miRNA-mRNA relationships are discovered to be significantly related to LIHC patient survival, as shown in Fig. 5.

By multivariate Cox proportional hazards regression analysis, we find that the high expression of NEAT1 and the low expression of LINC00311, C5orf17 are associated with the poor overall survival of LIHC patients. Interestingly, there is no significant association between the expression of three miRNAs and two mRNAs in ceRNAs, but the high expression of ceRNAs is related to poor overall survival of LIHC patients. This evidence illustrates that these lncRNAs may affect the expression of related miRNA and mRNA and then affects the expression of downstream molecules. It also suggests that these lncRNAs or ceRNAs may be regarded as prognostic-related signatures for LIHC. These above observations indicate that the ceRNA co- module identified by sparse ICA may be potential biomarkers for the survival of LIHC patients.

### E. Comparison with Other Algorithms

Various algorithms have been developed for CeRNA network construction methods, mainly based on the jNMF model, such as SNMNMF algorithm [32], MCNMF [55], MDJNMF algorithm [56] and NSOJNMF algorithm [57]. However, all of these algorithms use a variety of prior information to construct the objective function. Prior knowledge is considered to have the effect of driving the model decomposition within the model and improving the reliability of the results, and therefore cannot be fairly compared. In addition, models with multiple constraints are more dependent on the choice of parameters, and the random initialisation approach also causes the algorithms to be irreducible. Therefore, this paper compares the SparseICA algorithm with the JNMF model [58] without the use of prior knowledge, including the efficiency of the decomposition of different data, measured by the average correlation. Secondly, the time of model operations is calculated, as shown in TABLE 2.

**TABLE 2:** Comparison with Joint NMF Model

Different methods	Average correlation			Time cost
	X1_WH1	X2_WH2	X3_WH3	
JNMF	0.91	0.87	0.98	62s
Sparse ICA	0.87	0.88	0.99	1.4s

The number of components of JNMF in TABLE 2 is consistent with the settings of the SparseICA algorithm. From TABLE 2, it can be seen that the SICA algorithm used in this paper is weaker than JNMF in terms of model decomposition efficiency in general due to the JNMF algorithm, especially in lncRNA and mRNA, and in miRNA due to the fact that miRNA itself is more, and the sparsity constraint of SparseICA enhances the sparsity property of the miRNA matrix decomposition process. In terms of time cost, SparseICA is less time consuming in the same hardware environment.

## IV. Discussions

As a classical method for blind source separation, ICA is based on higher-order statistical moments, and its purpose is to decompose the observed multivariate data into the sources which are assumed statistically independent and non-Gaussian, and it has been successfully applied to the data analysis in various fields and to find the underlying independent sources. But as far as we know, it has not yet been used for the analysis of RNA expression data. Therefore, this is the first time for us to apply this method to the analysis of RNA expression, and the evaluation results show that the performance of this method is effective, and some significant results have obtained through it in the analysis of RNA data in patients with liver cancer.

Since the regulatory relationship between mRNA, miRNA and lncRNA in patients with liver cancer has its own specificity, thus presents a certain degree of independence, which is the reason that ICA is adopted in this paper. In addition to independence, current ICA studies also tend to consider sparsity. For liver cancer, the number of RNA elements regulated between mRNA, miRNA and lncRNA is small compared with the respective total number of RNA, so the module matrix obtained by the proposed sparse ICA method also presents certain sparsity. Therefore, the wavelet sparse analysis method is first used to conduct sparse approximation for the expression matrices of three types of RAN in the sparse ICA method.

Furthermore, in order to reduce the redundant information contained in the sample data and the size of the sample dimensions, as well as the complexity of subsequent calculations, PCA is first used to conduct dimensionality reduction processing on the data before the sparse approximation expression of sample data is carried out in this paper, and the dimension size after dimensionality reduction is determined according to the contribution rate of principal components in the PCA. In this study, the number of components with a contribution rate of more than 90% is selected as the dimension size after dimension reduction, and 40 principal components are selected in the results.

In addition, the APC algorithm was adopted to obtain the final co- modules of the three RNA from the modules under multiple running conditions in our study, and then using them for the selection of different RNA elements. Different from the traditional clustering methods, such as K-means which need to determine the number of clustering in advance, APC can determine the number of clusters adaptively. Therefore, it overcomes the problem of pre-determining the number of clustering in the traditional clustering method and reduces the adverse influence brought by human subjective factors. Two co- modules were obtained from the results of multiple runs by APC method in this study. In the process of selecting the significant expression elements of the three RNAs, the row co- modules corresponding to the module matrix S obtained by APC are z-scored by putting these three types of RNAs together in this study. In addition, they can also be z-scored separately, and show the same results when the three types of RNA are z-scored together. Therefore, only the results of one situation are presented in this study.

From the experimental results of the ceRNA construct, we found that most of the lncRNAs competed with mRNA to bind three miRNAs. This suggests that these miRNAs play a key role in the development of liver cancer. This is consistent with some existing descriptions in the literature. For example, among miRNAs, miR-25 is highly expressed at early stages and plays an important role in the progression of HCC, which may be of prognostic value and facilitate the development of novel therapeutic approaches for HCC [59]. miR-218 expression was significantly downregulated in HCC tissues and cell lines. Gain-of-function and loss-of-function experiments showed that forced expression of miR-218 in HCC cells inhibited cell migration/invasion and reversed epithelial-mesenchymal transition (EMT) to mesenchymal-epithelial transition (MET), whereas deletion of miR-218 promoted cell migration/invasion and contributed to the EMT phenotype [60]. Silencing of miR-218-5p inhibited activation of the JAK2/STAT3 pathway by targeting KLF9 [61], while KLF4, which belongs to the same family, could inhibit the JAK2/STAT3 pathway, which in turn affects cancer cell development [62]. Furthermore, most of the lncRNAs in the experimental results were found to be associated with liver disease, while the endogenous competitive relationships constructed by miRNA-centric had good discrimination in patient survival curves, suggesting that the SparseICA-based approach could be effective in constructing a ceRNA network for HCC and could serve as a potential indicator for predicting patient survival status.

In addition, there are still some shortcomings in this study, including the lack of data collection, the construction of ceRNA network not only includes lncRNA but also other RNA molecules such as circrna, and we need to include circrna molecules to improve the ceRNA network in future studies. At the same time, due to the limitations of the experimental equipment, biological validation of the experimental results was not performed in this paper, which needs to be done later. Finally, the method in this paper does not take into account the benefit of a priori knowledge to the extent that it cannot be fairly compared with current state-of-the-art ceRNA construction methods. At a later stage, we will investigate a priori knowledge-driven ICA methods to compare the current experimental results and improve the reliability and biological interpretability of the algorithm.

## V. Conclusion

In this paper, a new sparse ICA method was proposed to identify the co- module information contained in mRNA, miRNA and lncRNA, and applied it for the three RNA data analysis of LIHC patients. The results revealed that ceRNA includes 46 lncRNAs, 3 mRNAs, 3miRNAs, where all lncRNAs, mRNAs and 1 miRNA are significantly differentially expressed, and the elements of ceRNA constructed by co- module are closely associated with LIHC. Therefore, the ceRNA co- module identified by sparse ICA may be potential biomarkers for the survival of LIHC patients.
